# Spatial transcriptomics reveals immune remodeling in lymph nodes of gastric adenocarcinoma

**DOI:** 10.1007/s10585-026-10429-7

**Published:** 2026-09-17

**Authors:** Thanawat Suwatthanarak, Chawisa Nampoolsuksan, Pariyada Tanjak, Kullanist Thanormjit, Onchira Acharayothin, Amphun Chaiboonchoe, Tharathorn Suwatthanarak, Manop Pithukpakorn, Wipapat Vicki Chalermwai, Jirawat Swangsri, Asada Methasate, Vitoon Chinswangwatanakul, Thammawat Parakonthun

**Affiliations:** 1 Siriraj Cancer Center, Faculty of Medicine Siriraj Hospital, Mahidol University, Bangkok, Thailand; 2 Department of Surgery, Faculty of Medicine Siriraj Hospital, Mahidol University, Bangkok, Thailand; 3 Siriraj Upper Gastrointestinal Cancer Center, Department of Surgery, Faculty of Medicine Siriraj Hospital, Mahidol University, Bangkok, Thailand; 4 Department of Pharmacology, Faculty of Medicine Siriraj Hospital, Mahidol University, Bangkok, Thailand; 5 Department of Medicine, Faculty of Medicine Siriraj Hospital, Mahidol University, Bangkok, Thailand; 6 Department of Pathology, Faculty of Medicine Siriraj Hospital, Mahidol University, Bangkok, Thailand

**Keywords:** Gastric adenocarcinoma, Immune gene expression, Lymph node metastasis, Nodal burden, Spatial transcriptomics

## Abstract

Lymph node (LN) metastasis is a major determinant of prognosis in gastric cancer, yet the immune microenvironment of metastatic versus non-metastatic nodes remains incompletely defined. Spatial resolution of tumor–immune interactions may clarify mechanisms of immune escape associated with nodal progression. We performed compartment-resolved spatial transcriptomic profiling to characterize immune gene expression in gastric cancer LNs. Forty-seven formalin-fixed paraffin-embedded LNs from 13 patients with T2–T4, M0 gastric adenocarcinoma (N1–2: *n* = 7; N3: *n* = 6) were analyzed using the NanoString GeoMx Digital Spatial Profiler and an 84-gene immune pathways panel. Twenty-nine non-metastatic and 18 metastatic LNs were profiled. Regions of interest were segmented into tumor and immune compartments, yielding 65 spatially defined compartments. Differential expression was assessed across three comparisons: metastatic versus non-metastatic LNs; metastatic LNs from N1–2 versus N3 patients; and non-metastatic LNs from N1–2 versus N3 patients. Within the immune compartments, non-metastatic LNs showed higher expression of T-cell activation and checkpoint genes (CD3E, CD27, PDCD1, CTLA4), consistent with preserved immune surveillance. Metastatic LNs were enriched for WNT signaling and adhesion-related genes (CTNNB1, ITGAV) and epithelial markers (EPCAM), indicating tumor-driven immune remodeling. N3 metastatic LNs demonstrated increased ICOSLG, IL6, and IFNGR1 expression, suggesting chronic inflammatory activation and immune dysfunction. Notably, non-metastatic LNs from N3 patients upregulated antigen-presentation genes (CD74, HLA-DRB), consistent with early immune conditioning. No significant differences emerged in tumor compartments or pseudo-bulk analyses. Spatial transcriptomic profiling reveals nodal burden–associated immune remodeling, and identifies candidate biomarkers and therapeutic targets for precision immunotherapy in gastric cancer.

**Supplementary Information:**

The online version contains supplementary material available at https://doi.org/10.1007/s10585-026-10429-7.

## Introduction

 Gastric cancer is a leading cause of cancer-related mortality globally, and lymph node (LN) metastasis remains one of the most important prognostic indicators [1–3]. The number and anatomic distribution of metastatic LNs directly influence TNM staging, surgical planning, and perioperative chemotherapy decisions [4, 5]. Although the clinical significance of nodal involvement is well established, the biological processes that enable immune escape and support metastatic colonization within LNs remain poorly defined, particularly in gastric cancer.

LN evaluation in gastric cancer has traditionally relied on histopathologic detection of tumor cells [4, 5]; however, this approach fails to capture broader tumor–immune interactions within the lymphoid microenvironment. LNs are not passive filters for disseminated cancer cells—they are active immunologic organs responsible for antigen presentation, lymphocyte activation, and immune surveillance [6]. These functions can be disrupted during cancer progression, leading to immune suppression, immune exclusion, or pre-metastatic niches that facilitate tumor spread [6].

Recent studies in other solid tumors have highlighted immune-cell dysfunction, checkpoint activation, and inflammatory cytokine signaling within tumor-draining LNs [7, 8]. However, most rely on bulk transcriptomics or protein-level analyses that lack spatial context [7, 8]. Bulk analysis averages signals across tumor and stromal regions, potentially obscuring localized immune interactions and compartment-specific gene expression [9]. In metastatic LNs, this limitation is particularly problematic because immune responses may be spatially restricted or masked by tumor-dominant transcriptional activity.

Spatial transcriptomic technologies now enable high-resolution interrogation of these processes [10, 11]. Among them, the NanoString GeoMx Digital Spatial Profiling (DSP) performs transcript-level profiling of formalin-fixed paraffin-embedded (FFPE) tissues via immunofluorescence-guided selection of regions of interest (ROIs) [11, 12]. By segmenting tissue into epithelial and immune compartments, this platform enables examination of gene expression patterns specific to tumor cells and the immune microenvironment within the same anatomic region [11, 12].

**Scheme 1:**
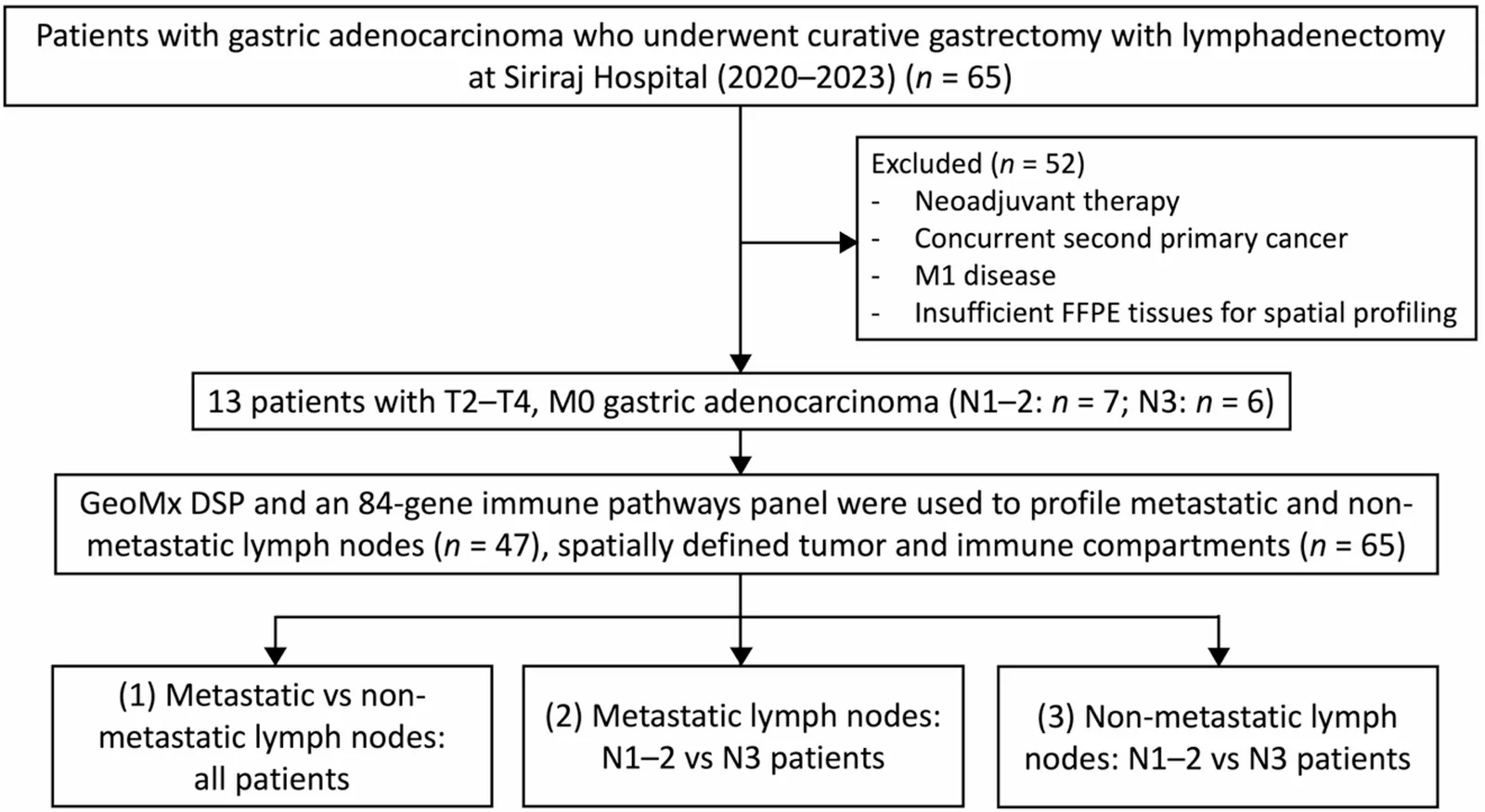
Study design and analytical workflow. Of 65 patients who underwent curative gastrectomy with lymphadenectomy at Siriraj Hospital (2020–2023), 13 patients with T2–T4, M0 gastric adenocarcinoma (N1–2: *n* = 7; N3: *n* = 6) met the inclusion criteria. Patients were excluded for neoadjuvant therapy, concurrent second primary malignancies, M1 disease, or insufficient FFPE tissue. The 2020–2023 timeframe was selected to ensure the preservation and suitability of FFPE blocks for GeoMx DSP analysis. A total of 47 lymph nodes (18 metastatic and 29 non-metastatic) were profiled using an 84-gene immune pathways panel, yielding 65 spatially defined tumor and immune compartments. Differential expression analyses compared: (1) metastatic vs. non-metastatic lymph nodes across all patients; (2) metastatic lymph nodes from N1–2 vs. N3 patients; and (3) non-metastatic lymph nodes from N1–2 vs. N3 patients. Abbreviations: DSP, digital spatial profiling; FFPE, formalin-fixed paraffin-embedded

In this study, we used GeoMx DSP and an 84-gene immune pathways panel to profile metastatic and non-metastatic LNs from 13 patients with T2–T4, M0 gastric adenocarcinoma (Scheme 1). We analyzed gene expression within spatially defined tumor and immune compartments across three comparisons (Scheme 1). These comparisons were metastatic vs. non-metastatic LNs, metastatic LNs from patients with intermediate (N1–2) vs. advanced (N3) nodal burden, and non-metastatic LNs from N1–2 vs. N3 patients (Scheme 1). These comparisons were designed to delineate compartment-specific immune alterations associated with nodal metastasis and increasing tumor burden and to assess potential systemic or pre-metastatic immune changes in uninvolved LNs. To our knowledge, spatial transcriptomic profiling of metastatic LNs in gastric cancer remains extremely limited, and compartment-specific characterization of immune gene expression has not been previously reported. This work offers novel insights into the immune architecture of regional metastases and may provide a foundation for identifying spatially defined biomarkers of immune escape, prognostication, and therapeutic targeting in gastric cancer.

## Materials and methods

### Patient cohort and tissue preparation

Patients with gastric adenocarcinoma who underwent curative gastrectomy with lymphadenectomy at the Faculty of Medicine Siriraj Hospital, Bangkok, Thailand, between 2020 and 2023 (*n* = 65) were screened for eligibility. Cases treated between 2020 and 2023 were selected to ensure adequate preservation and suitability of FFPE tissue blocks for GeoMx DSP analysis. Patients were excluded if they had received neoadjuvant therapy, had concurrent second primary malignancy, presented with M1 disease, or had insufficient FFPE tissue for spatial profiling (*n* = 52). Thirteen patients met inclusion criteria and were included in the final cohort. All were pathologically staged as T2–T4, M0 and classified according to the American Joint Committee on Cancer (AJCC) TNM system as N1–2 (*n* = 7) or N3 (*n* = 6). Cases were selected to represent a spectrum of nodal burden. A total of 47 FFPE LN specimens (metastatic and non-metastatic) were available for spatial transcriptomic analysis.

For each patient, metastatic and non-metastatic LNs from lymphadenectomy stations (D1–D2) were identified by a gastrointestinal pathologist via routine hematoxylin and eosin review. Representative LNs were selected with priority given to blocks with intact morphology and adequate tumor or immune-rich regions suitable for spatial profiling. Clinical data, including age, sex, tumor location, TNM stage, and nodal yield, were recorded, and all specimens were anonymized before analysis. The total number of examined LNs ranged from 28 to 109 per patient. A total of 47 LNs (18 metastatic and 29 non-metastatic) were selected for GeoMx DSP analysis. The profiled LNs originated from D1 stations (1, 3, 4, 5, 6, and 7) and D2 stations (8a, 9, 10, 11p, and 12a), as defined by the Japanese Gastric Cancer Treatment Guidelines. Detailed information regarding LN yield, metastatic LN count, and anatomical nodal stations is provided in Supplementary Tables S1 and S2.

Cores measuring 3 mm were extracted from selected FFPE LN blocks and assembled into tissue microarrays using a manual tissue microarrayer. Core selection was guided by a gastrointestinal pathologist to ensure inclusion of regions with intact morphology and representative tumor and immune compartments.

The study protocol was approved by the Siriraj Institutional Review Board (COA no. Si 389/2023). The study covered retrospective and prospective cases between 2020 and 2023, and all procedures complied with the Declaration of Helsinki.

### GeoMx digital spatial profiling

Spatial transcriptomic profiling was performed on LN tissues using the NanoString GeoMx DSP platform with the GeoMx Immune Pathways Panel, comprising 84 immune-related genes (Bruker Spatial Biology, Inc., Seattle, WA, USA). FFPE LN sections were cut at 5 μm and mounted on positively charged glass slides before being deparaffinized, rehydrated, and subjected to antigen retrieval per the manufacturer’s guidelines (GeoMx DSP Manual Slide Preparation, MAN-10150-03). To enable spatial profiling of tumor and immune regions, tissue sections were stained with SYTO13 for nuclear visualization, an anti-pan-cytokeratin (PanCK) antibody to identify epithelial/tumor cells, and an anti-CD45 antibody to highlight leukocyte-rich areas using reagents provided in the GeoMx Morphology Kit (Bruker Spatial Biology, Inc., Seattle, WA, USA). These immunofluorescence markers were used to guide ROI selection, while compartment segmentation was performed based on PanCK-positive and PanCK-negative regions for GeoMx DSP analysis.

The slides were scanned on the GeoMx DSP system (Bruker Spatial Biology, Inc.), and ROIs were manually selected based on morphology and immunofluorescence signals. ROIs were chosen to capture representative tumor-infiltrated regions and adjacent immune-rich areas within each LN while avoiding tissue artifacts, necrosis, and poorly preserved regions. All analyzed LN tissues were incorporated into tissue microarrays using standardized 3-mm cores. ROIs were then segmented into tumor (PanCK⁺) and immune (PanCK⁻) compartments using GeoMx software (version 3.1.0.222). One ROI was selected per LN, and all ROIs were standardized to identical dimensions (660 μm × 785 μm). To minimize potential bias related to tissue sampling size, comparisons were performed using compartment-specific expression profiles derived from these standardized ROIs rather than from entire LNs. In addition, the segmented PanCK-positive area was quantified to assess potential differences in profiled tumor burden between groups.

After segmentation, ultraviolet light photocleaved barcoded oligonucleotide probes within each compartment. Released oligonucleotides were collected via microcapillary and hybridized to fluorescent optical barcodes using the NanoString nCounter assay (GeoMx DSP nCounter Readout User Manual, MAN-10089-09). The gene counts were generated for 84 targets within each spatially defined compartment.

The gene counts were imported into the GeoMx DSP Analysis Suite (version 3.1.0.222) for quality control, which assessed probe performance, detection thresholds vs. negative controls, signal distributions, and percent probe coverage. Qualified ROIs were normalized to the geometric mean of housekeeping genes OAZ1, UBB, RAB7A, SDHA, and POLR2A following standard reference-based normalization for GeoMx DSP data.

### Statistical analysis

Differential expression analysis was performed in the GeoMx DSP Analysis Suite (version 3.1.0.222). Three primary comparisons assessed transcriptional differences across spatial compartments: (1) metastatic vs. non-metastatic LNs in immune compartments; (2) metastatic LNs from N1–2 vs. N3 patients across immune, tumor, and pseudo-bulk compartments; and (3) non-metastatic LNs from N1–2 vs. N3 patients in immune compartments. Statistical significance used a false discovery rate < 0.05 with Benjamini–Hochberg adjustment. Gene annotations followed the NanoString Immune Pathways panel specifications. Recurrence-free survival was analyzed with the log-rank test in GraphPad Prism version 8 (GraphPad Software Inc., San Diego, CA, USA). Clinicopathologic characteristics were compared using Fisher’s exact test for categorical variables and the Mann–Whitney *U* test for continuous variables in IBM SPSS Statistics version 31 (IBM Corp, Armonk, NY, USA). A *P* value < 0.05 was considered statistically significant.

### Data visualization

Kaplan–Meier curves and outputs from the GeoMx DSP Analysis Suite, including volcano plots, were visualized in GraphPad Prism version 8. A heatmap was generated using NG-CHM Builder (https://build.ngchm.net/NGCHM-web-builder/).

## Results

### Patient cohort and spatial profiling overview

Thirteen patients with T2–T4, M0 gastric adenocarcinoma were included: 7 with N1–2 and 6 with N3 nodal status per the AJCC staging system. For each patient, FFPE LNs were profiled on the NanoString GeoMx DSP using an 84-gene immune pathways panel. Both metastatic and non-metastatic nodes were examined; ROIs were segmented into tumor and immune compartments. Forty-seven high-quality ROIs and 65 compartments met quality-control criteria and entered the final analysis. DEG analyses addressed three contrasts. These were metastatic vs. non-metastatic LNs in immune compartments; metastatic LNs from N1–2 vs. N3 patients across immune, tumor, and pseudo-bulk compartments; and non-metastatic LNs from N1–2 vs. N3 in immune compartments. Table 1 summarizes clinicopathologic characteristics by nodal stage (N1–2 vs. N3). The N3 cohort was significantly associated with diffuse tumor location, whereas N1–2 cases were predominantly localized. Pathologic T stage distribution was comparable between groups. However, N3 patients demonstrated higher frequencies of lymphovascular invasion (LVI) and perineural invasion (PNI), consistent with more aggressive tumor biology.

**Table 1:** Clinicopathologic characteristics by nodal stage (N1–2 vs. N3) in gastric adenocarcinoma

Characteristics	N1-2	N3	*P* value*
Number of patients	7	6	–
*Sex*			0.559
Male	3 (42.9%)	1 (16.7%)	
*Age*			0.152
Median, IQR (y)	72, 56–79	60.5, 57–63	
*Pathological T stage*			1.000
pT3	5 (71.4%)	5 (83.3%)	
pT4	2 (28.6%)	1 (16.7%)	
*Pathological N stage*			**< 0.001**
pN1-pN2	7 (100%)	0 (0%)	
pN3	0 (0%)	6 (100%)	
*LVI*			0.266
Positive LVI	3 (42.9%)	5 (83.3%)	
*PNI*			0.462
Positive PNI	5 (71.4%)	6 (100%)	
*Tumor site*			**0.018**
Localized (antrum/pylorus/lesser curvature)	6 (85.7%)	1 (16.7%)	
Diffuse (body/fundus/cardia/multiple sites)	1 (14.3%)	5 (83.3%)	
*Differentiation*			0.462
Poor	7 (100%)	5 (83.3%)	
Moderate	0 (0%)	1 (16.7%)	
*Adjuvant CMT*			0.592
No	1 (14.3%)	2 (33.3%)	
Complete	4 (57.1%)	2 (33.3%)	
Incomplete	2 (28.6%)	2 (33.3%)	
*Number of profiled LNs*			–
Metastatic LNs	7	11	
Non-metastatic LNs	16	13	

Note: Values are *n* (%) unless indicated. Age is median, IQR in y. Pathologic categories follow the American Joint Committee on Cancer TNM system. Bold values indicate statistical significance.

*P values were calculated with the Fisher exact test (categorical) and the Mann–Whitney *U* test (continuous).

CMT, chemotherapy; LN, lymph node; LVI, lymphovascular invasion; pM, pathologic M category; pN, pathologic N category; PNI, perineural invasion; pT, pathologic T category; IQR, interquartile range; y, years.

### Recurrence-free survival of gastric cancer patients stratified by nodal status

**Fig. 1:**
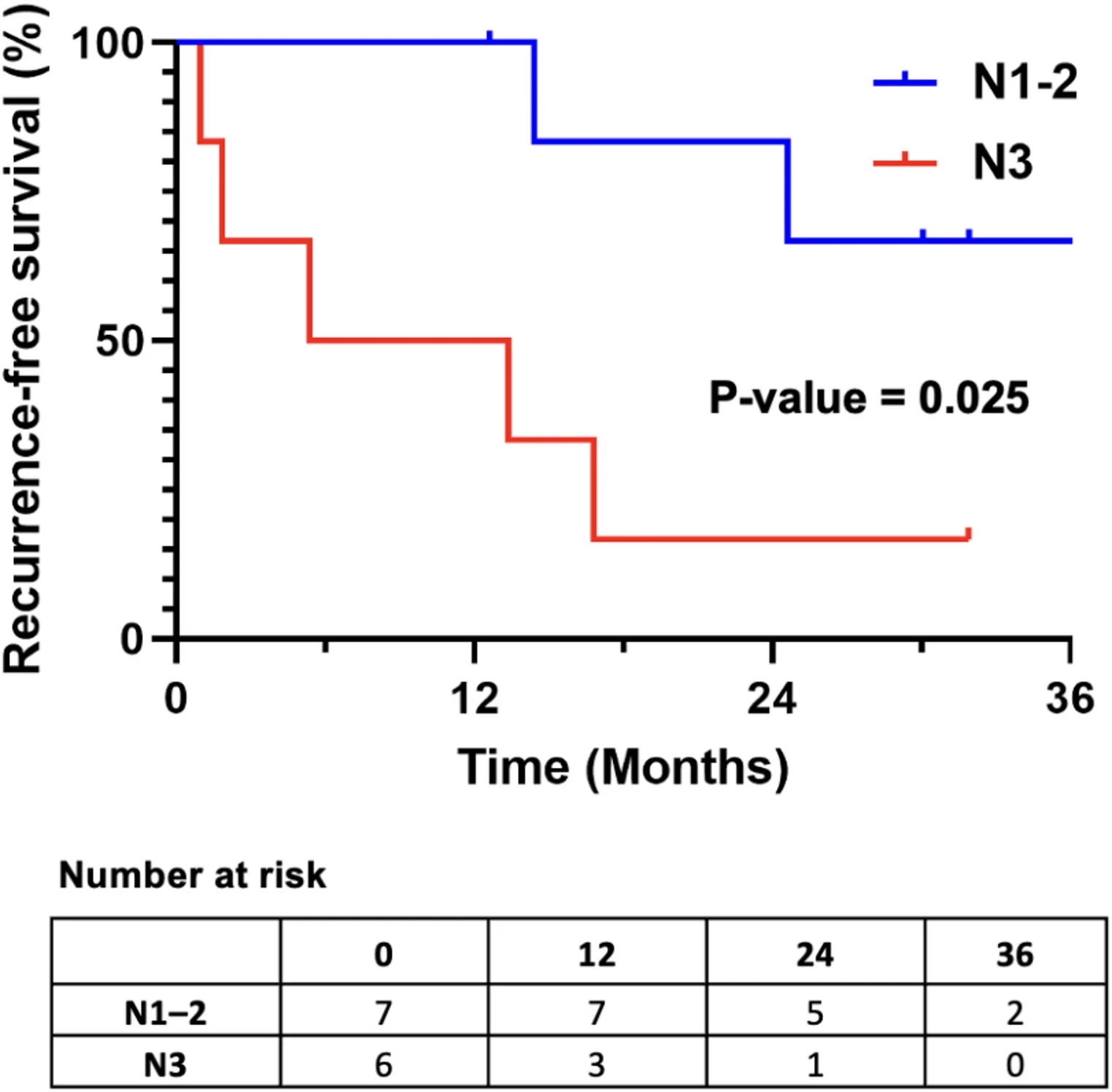
Recurrence-free survival in gastric cancer patients stratified by nodal status. Kaplan–Meier curve comparing recurrence-free survival between patients with N1–2 (*n* = 7, blue) and N3 (*n* = 6, red) lymph node involvement. Patients with N3 nodal status exhibited significantly worse recurrence-free survival compared to those with N1–2 disease (*P* = 0.025, log-rank test)

To assess the clinical relevance of LN burden, we compared recurrence-free survival between N1–2 and N3 groups. Kaplan–Meier analysis showed significantly shorter recurrence-free survival in N3 vs. N1–2 (log-rank, *P* = 0.025; Fig. 1). These findings reinforce the prognostic impact of extensive nodal metastasis and motivate immune profiling in relation to nodal stage.

### Spatial segmentation and profiling of tumor and immune compartments in LNs

**Fig. 2:**
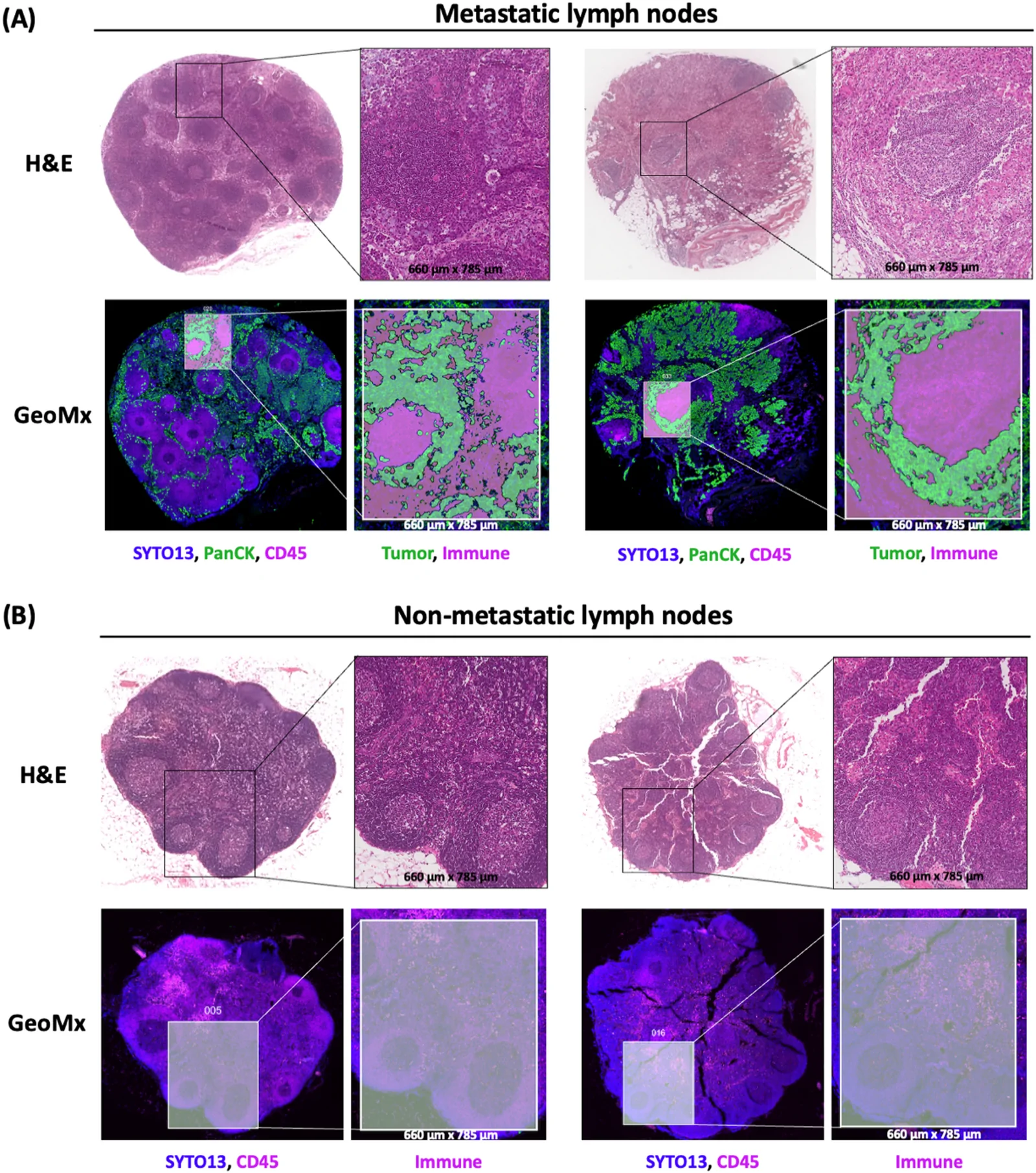
Representative histological and GeoMx DSP images of metastatic and non-metastatic lymph nodes in gastric adenocarcinoma. (**A**) Metastatic lymph nodes with hematoxylin and eosin (H&E) staining (top) and corresponding GeoMx immunofluorescence images (bottom). PanCK (green) identifies tumor cells, CD45 (magenta) marks immune cells, and SYTO13 (blue) labels nuclei. Tumor and immune compartments were segmented for spatial profiling. (**B**) Non-metastatic lymph nodes with H&E (top) and GeoMx images (bottom). ROIs (660 μm × 785 μm) were selected and segmented for downstream transcriptomic analysis

Using GeoMx DSP, we profiled the selected regions from metastatic and non-metastatic LNs (Fig. 2). Metastatic LNs displayed distinct tumor areas surrounded by immune populations, enabling precise compartmental segmentation into tumor and immune zones (Fig. 2A). Non-metastatic LNs showed abundant immune cells without detectable tumor infiltration (Fig. 2B). Representative H&E and immunofluorescence images confirmed compartment integrity and guided ROI selection for analysis (Fig. 2). To assess whether differences in transcriptional profiles could be attributed to variation in profiled tumor burden, we compared the segmented PanCK-positive area between metastatic LNs from N1–2 and N3 patients. No significant difference was observed between the two groups (*P* = 0.536; Supplementary Figure S1), suggesting that the identified transcriptional differences were unlikely to be driven by variation in the size of the profiled tumor compartment. This spatial resolution enabled targeted gene-expression profiling within histologically distinct compartments, facilitating downstream comparisons of immune activity and tumor–immune interactions across nodal types and disease burden.

**Fig. 3:**
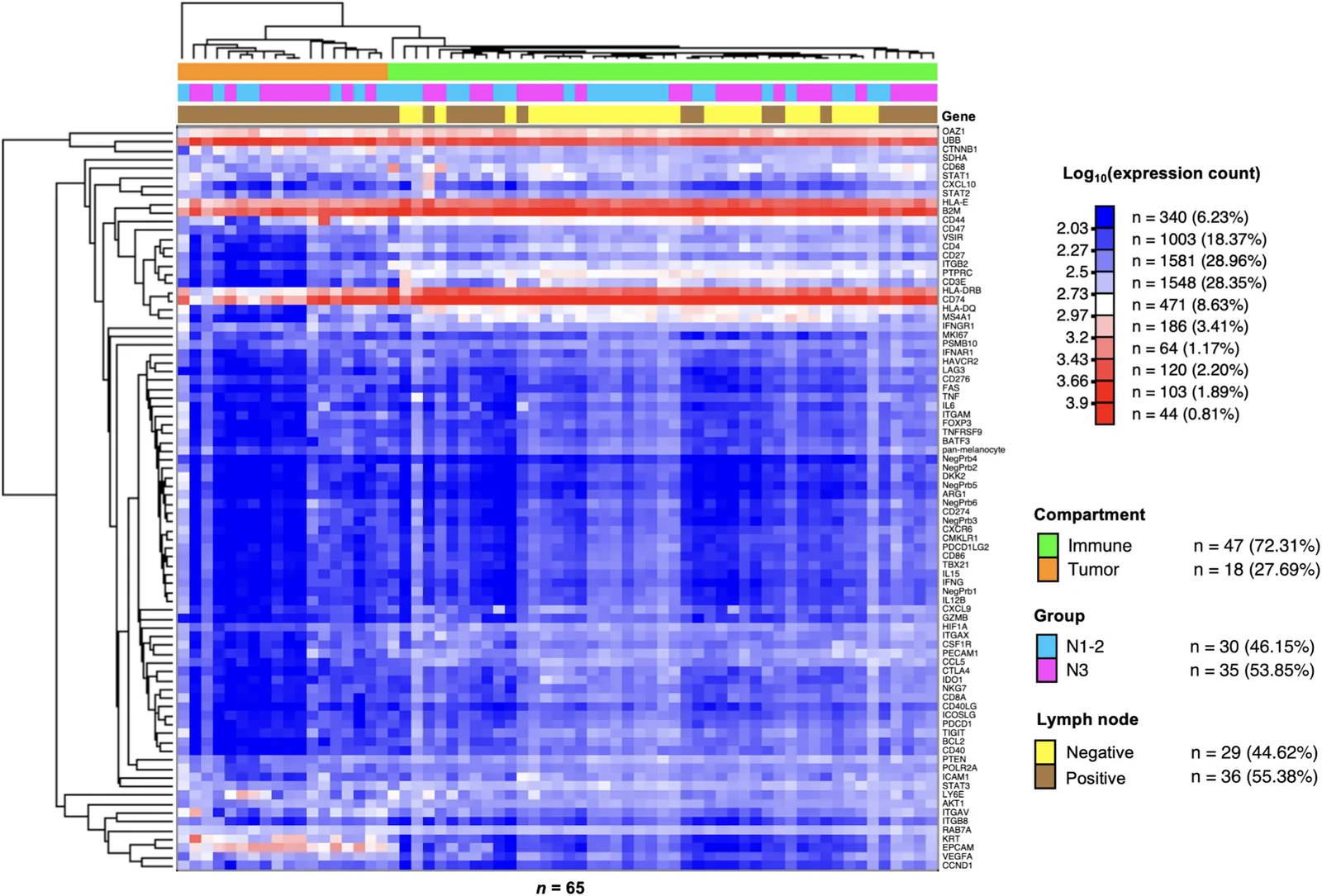
Gene-expression heatmap across all compartments. Unsupervised hierarchical clustering of all normalized gene counts across tumor and immune compartments demonstrates two clearly separated clusters corresponding to compartment type. Immune compartments show uniformly high expression of lymphoid- and myeloid-associated genes, whereas tumor compartments display low immune-gene expression with selective upregulation of tumor-intrinsic markers. Annotation bars indicate compartment type, nodal stage, and metastatic status. The clustering pattern is consistent with biologically distinct transcriptional programs and supports the rationale for compartment-specific downstream analyses

As shown in Fig. 3, unsupervised hierarchical clustering of the normalized transcript counts across all spatially defined compartments demonstrates segregation into tumor and immune clusters. Immune compartments show enrichment of lymphoid- and myeloid-associated transcripts, whereas tumor compartments display lower immune gene expression with selective upregulation of tumor-associated markers. Annotation bars indicate compartment type, nodal stage, and metastatic status. The observed clustering pattern is consistent with biologically distinct transcriptional programs and supports the validity of compartment-specific analyses. Principal component analysis of all 65 profiled compartments revealed distinct clustering of tumor (PanCK⁺) and immune (PanCK⁻) compartments (Supplementary Figure S2). The separation was primarily driven by PC1 (50.9% variance explained) and PC2 (15.2% variance explained), supporting the biological distinction between tumor and immune microenvironments captured by the GeoMx DSP platform.

### Comparison of immune compartments in metastatic and non-metastatic LNs: N1–3 gastric cancer patients

**Fig. 4:**
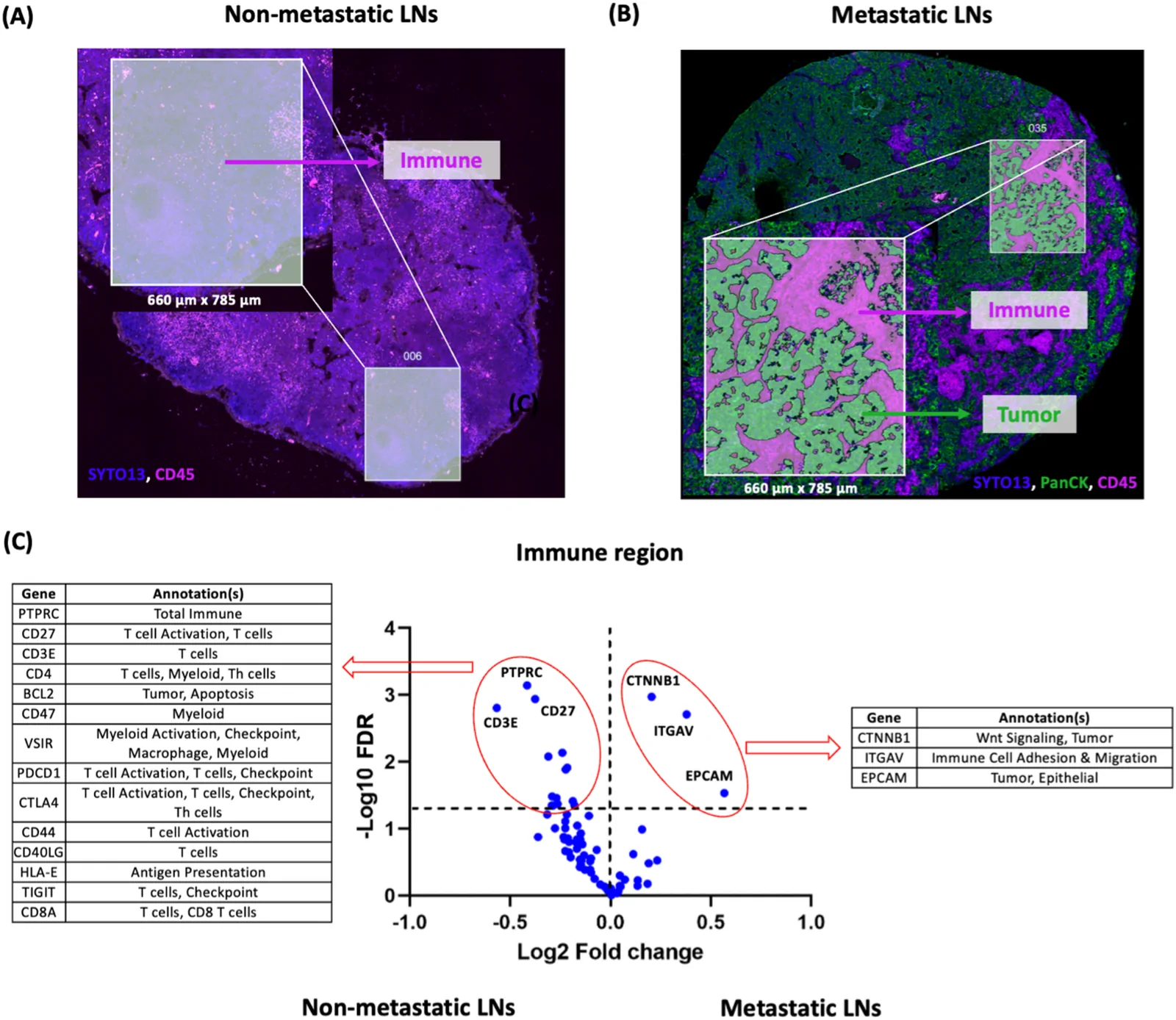
Comparison of differential gene expression in the immune compartment between non-metastatic and metastatic lymph nodes from N1–3 gastric cancer patients. (**A**) Representative GeoMx DSP ROI from a non-metastatic lymph node showing the immune-rich compartment. (**B**) Representative ROI from a metastatic lymph node showing compartment segmentation into tumor and immune regions. All ROIs measured 660 × 785 μm. Blue, SYTO13; green, PanCK; magenta, CD45. (**C**) Volcano plot showing differentially expressed genes in immune regions of interest from non-metastatic (*n* = 29) versus metastatic (*n* = 18) lymph nodes. Genes enriched in non-metastatic lymph nodes (left side) include markers of T cell activation and immune surveillance (e.g., PTPRC, CD27, CD3E, PDCD1, CTLA4), while metastatic lymph nodes (right side) show upregulation of tumor-associated and immune-suppressive markers (e.g., CTNNB1, ITGAV, EPCAM). Annotated gene panels highlight biological functions associated with each group, including T cell signaling, immune checkpoints, and Wnt/tumor pathways. The top three most significantly upregulated genes in each group are labeled. Abbreviations: FDR, false discovery rate; LN, lymph node; ROI, region of interest; DSP, digital spatial profiling

To assess how the immune microenvironment differs with metastasis, we compared differential expression within immune compartments from metastatic vs. non-metastatic LNs across all patients (Fig. 4A, B). Non-metastatic LNs showed higher expression of genes linked to T-cell abundance and activation, including CD3E, CD4, CD8A, CD27, and CD40LG (Fig. 4C) [13–15]. Immune checkpoint genes—PDCD1, CTLA4, TIGIT, and VSIR—were also upregulated, consistent with immunologically active yet regulated T-cell populations (Fig. 4C) [16, 17]. Additional upregulated genes included HLA-E and the apoptosis-regulating molecule BCL2, suggesting preservation of immune cell survival and immune activity (Fig. 4C) [18, 19].

In contrast, metastatic LNs were enriched for tumor-related and immune-evasion programs, with increased CTNNB1, ITGAV, and EPCAM expression (Fig. 4C) [20, 21]. These patterns indicate that immune compartments within metastatic nodes are dominated by tumor-derived signals, reflecting remodeling toward immunosuppression. Table 2 summarizes these contrasts. Non-metastatic nodes are immune-active and surveillance-competent, potentially responsive to immune checkpoint blockade (Table 2). Metastatic nodes are immune-suppressed and tumor-dominated, which may limit benefit from checkpoint blockade alone and justify combinations targeting Wnt, IL6, or CD47 pathways (Table 2). Together, these results underscore the relevance of nodal immune profiling for predicting therapeutic response and informing biomarker development in gastric cancer.

**Table 2:** Immune landscape and clinical relevance in immune compartments of non-metastatic vs. metastatic lymph nodes from N1–3 gastric cancer patients

Immune region	Non-metastatic lymph nodes	Metastatic lymph nodes
Immune status	Immune-active/surveillance-competent	Immune-suppressed/tumor-dominated
Immune activity	High (Rich in CD3E, CD8A, PDCD1, CTLA4, CD27 → active immune surveillance and checkpoint expression)	Low (Dominated by tumor markers like EPCAM, CTNNB1 and lacking cytotoxic immune genes → immunologically cold)
Clinical relevance	May indicate responsiveness to immune checkpoint blockade (e.g., anti–PD-1, anti–CTLA-4)	May exhibit limited response to ICB monotherapy
	Supports preserved immune function in early-stage disease	Potential benefit from combination strategies targeting Wnt signaling, IL6, or CD47-mediated immune evasion

Note: Parenthetical gene examples are illustrative of transcript enrichment and are not exhaustive.

ICB, immune checkpoint blockade; Wnt, wingless/integrated signaling pathway.

### Comparison of tumor and immune compartments in metastatic LNs: N1–2 vs. N3 gastric cancer patients

**Fig. 5:**
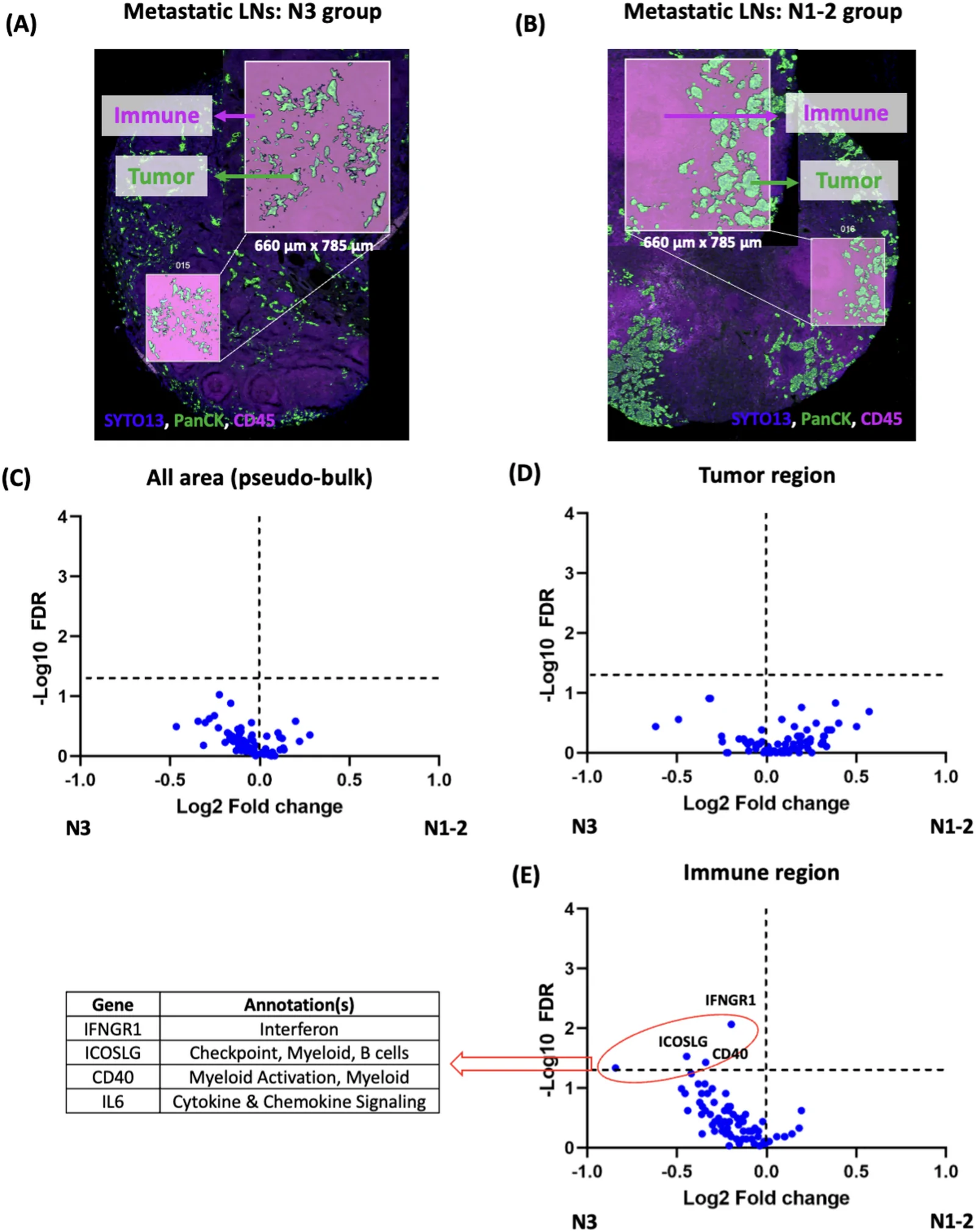
Spatial transcriptomic analysis of tumor and immune compartments in metastatic lymph nodes from N1–2 vs. N3 gastric cancer patients. The analysis revealed immune activation and dysfunction signatures enriched in metastatic lymph nodes of N3 gastric cancer patients. (**A**) Representative GeoMx DSP ROI from a metastatic lymph node of an N3 patient showing compartment segmentation into tumor and immune regions. (**B**) Representative ROI from a metastatic lymph node of an N1–2 patient. All ROIs measured 660 × 785 μm. Blue, SYTO13; green, PanCK; magenta, CD45. (**C**) Volcano plot of differentially expressed genes in the pseudo-bulked GeoMx area (combined tumor and immune compartments), comparing N3 (*n* = 22) versus N1–2 (*n* = 14). (**D**) Tumor compartment shows limited differential gene expression (N3: *n* = 11; N1–2: *n* = 7). (**E**) Immune region displays upregulation of IFNGR1, ICOSLG, CD40, and IL6 in N3 nodes, indicating interferon signaling, immune checkpoint activation, myeloid involvement, and inflammatory cytokine expression (N3: *n* = 11; N1–2: *n* = 7). These alterations support the presence of a chronically inflamed and immune-dysfunctional microenvironment in high-burden lymph node metastasis. The top three most significantly upregulated genes are labeled. Abbreviations: FDR, false discovery rate; LN, lymph node; ROI, region of interest; DSP, digital spatial profiling

To test whether the extent of nodal metastasis shapes local transcriptional programs, we compared metastatic LNs from N1–2 vs. N3 patients by pseudo-bulk, tumor-specific, and immune-specific analyses (Fig. 5A, B). No significant differentially expressed genes were detected in pseudo-bulk or tumor compartments between stages (Fig. 5C, D). This suggests a relatively stable epithelial transcriptome across nodal burden or, alternatively, masking of immune shifts in bulk profiles.

In contrast, the immune compartments revealed stage-specific immune dysregulation (Fig. 5E). IFNGR1, ICOSLG, CD40, and IL6 were significantly upregulated in N3 vs. N1–2 metastatic LNs, implicating enhanced interferon gamma signaling, costimulation, and inflammatory cytokine production (Fig. 5E) [22–24]. These signatures reflect a chronically inflamed, dysfunctional environment characterized by persistent stimulation, myeloid activation, and possible T-cell exhaustion. Table 3 summarizes these findings and suggests therapeutic hypotheses. Patients with N3 disease may benefit from combination immunotherapy targeting PD-1, IL6, or ICOSLG; these expression profiles may also mark immune exhaustion or poor prognosis (Table 3).

**Table 3:** Immune landscape and clinical relevance in immune compartments of metastatic lymph nodes: patients with N1–2 vs. N3

Immune region of metastatic lymph nodes	N1–2 group	N3 group
Immune status	N/A	Chronically inflamed/immune dysfunctional (Signs of exhaustion, inflammation, tolerogenic signaling)
Immune activity	N/A	Intermediate (Inflammation without effective T cell engagement; dysfunctional immune activity)
Clinical relevance	–	May benefit from combination immunotherapy (e.g., anti-PD-1 + IL6 or ICOSLG blockade)
		May serve as potential biomarkers for poor prognosis/immune exhaustion
		Myeloid-targeted therapies (e.g., anti-CD40, anti-IL6) may help restore immune balance

Note: Findings reflect compartment-specific signals in immune regions. “N/A” indicates not applicable.

### Comparison of immune compartments in non-metastatic LNs: N1–2 vs. N3 gastric cancer patients

**Fig. 6:**
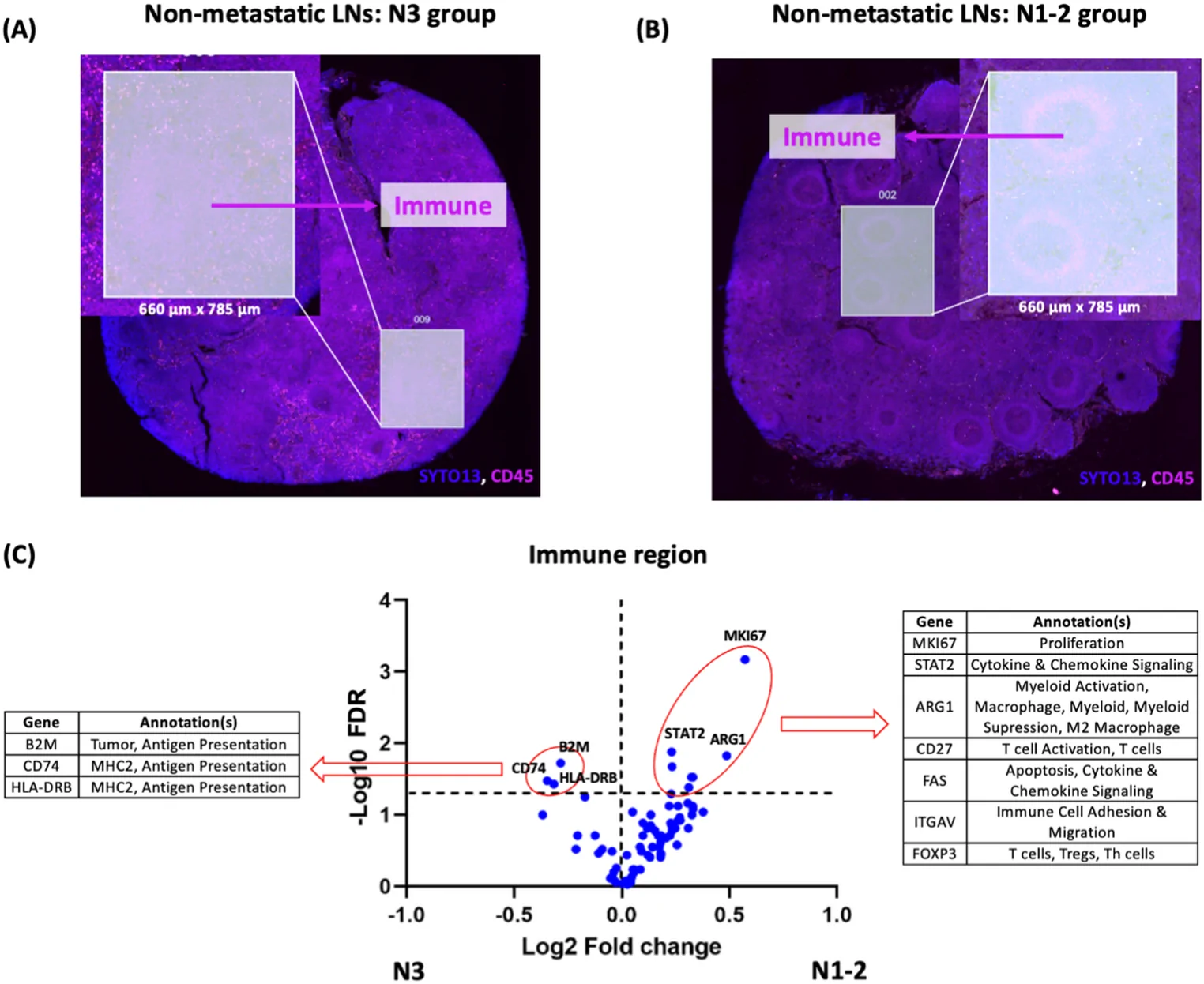
Differential gene expression analysis in the immune region of non-metastatic lymph nodes from N1–2 vs. N3 gastric cancer patients. (**A**) Representative GeoMx DSP ROI from a non-metastatic lymph node of an N3 patient showing the immune-rich compartment. (**B**) Representative ROI from a non-metastatic lymph node of an N1–2 patient. All ROIs measured 660 × 785 μm. Blue, SYTO13; magenta, CD45. (**C**) Volcano plot comparing gene expression in immune regions of non-metastatic lymph nodes between N3 (*n* = 13) and N1–2 (*n* = 16). Genes upregulated in N3 (e.g., B2M, CD74, HLA-DRB) are associated with antigen presentation and MHC class II signaling, while genes upregulated in N1–2 (e.g., MKI67, STAT2, ARG1, CD27, FOXP3) relate to proliferation, myeloid activation, T cell function, and immune regulation. The top three most significantly upregulated genes in each group are labeled. Abbreviations: FDR, false discovery rate; LN, lymph node; ROI, region of interest; DSP, digital spatial profiling

To examine systemic immune modulation before overt nodal metastasis, we evaluated immune compartments from non-metastatic LNs in N1–2 vs. N3 patients (Fig. 6A, B). Even without tumor infiltration, non-metastatic LNs from N3 patients upregulated B2M, CD74, and HLA-DRB—key mediators of antigen processing and MHC class II presentation (Fig. 6C) [25, 26]. These genes are typically induced by chronic immune stimulation, suggesting persistent activation or pre-metastatic remodeling.

By contrast, non-metastatic LNs from N1–2 patients showed broader, more balanced immune programs. Upregulated transcripts included MKI67 (proliferation), STAT2 (interferon signaling), CD27 (T-cell costimulation), FOXP3 (regulatory function), FAS (apoptosis), and ARG1 (myeloid modulation; Fig. 6C) [27–31]. This pattern indicates coexistence of effector, regulatory, and proliferative states within uninvolved nodes, consistent with a balanced yet active immune milieu.

Overall, N1–2 patients appear to retain functional surveillance in tumor-free LNs, whereas N3 patients display early immune exhaustion or remodeling. This suggests a systemic shift toward suppression that may precede metastasis and underscores the value of profiling uninvolved LNs to refine prognostic models and guide immunotherapy (Table 4). These features may provide early biomarkers of progression or immune escape (Table 4).

To further explore whether immune remodeling is detectable before overt LN metastasis, we performed an exploratory analysis comparing histologically negative LNs from node-negative (N0) patients (*n* = 4, Supplementary Table S3) with non-metastatic LNs from node-positive (N1–N3) patients. Differential expression analysis identified four significantly altered genes, including IFNGR1, HIF1A, CTNNB1, and PTPRC (Supplementary Figure S3). These findings suggest that transcriptional alterations may already be present in tumor-draining LNs prior to histologically detectable metastatic involvement, further supporting the concept of early nodal immune remodeling.

**Table 4:** Immune landscape and clinical relevance in immune compartments of non-metastatic lymph nodes: patients with N1–2 vs. N3

Immune region of non-metastatic lymph nodes	N1–2 group	N3 group
Immune status	Immune-regulated/balanced surveillance (Proliferative, tolerogenic, but responsive)	Pre-metastatic conditioning (Chronic antigen presentation; immune stimulation or dysfunction)
Immune activity	Intermediate–high (Mixed activation/suppression; functional immune presence)	Intermediate (Antigen-presenting but lacks strong T-effector or cytotoxic signals)
Clinical relevance	• May be responsive to ICB, as checkpoint and effector signals co-exist • Presence of FOXP3 and ARG1 suggests regulatory/suppressive populations and warrants caution	• May reflect early immune remodeling or dysfunction • May serve as biomarkers of immune escape or metastatic risk • Supports the inclusion of uninvolved LNs in spatial biomarker panels

Note: Parenthetical descriptors summarize representative functional programs inferred from expression patterns in immune regions.

ICB, immune checkpoint blockade; LN, lymph node.

## Discussion

This study provides a spatial transcriptomic analysis of LNs in gastric adenocarcinoma using the NanoString GeoMx DSP platform. Independent profiling of tumor and immune compartments revealed distinct expression patterns associated with metastasis and nodal burden, indicating progressive, spatially localized immune remodeling within the lymphatic microenvironment.

Non-metastatic LNs displayed a transcriptionally active immune landscape enriched for T cell activation genes (CD3E, CD4, CD8A, CD27), costimulatory molecules (CD40LG, CD44), and immune checkpoints (PDCD1, CTLA4, TIGIT). These features suggest preserved immunologic competence and potential responsiveness to ICB. They align with data in gastrointestinal cancers showing that tertiary lymphoid structures and antigen-presenting cells in uninvolved nodes correlate with favorable outcomes [32–34]. Clinically, immune-active LNs may serve as biomarkers for early-stage disease and inform timing or use of immunotherapy in localized settings.

By contrast, metastatic LNs showed decreased immune activation with upregulation of tumor-intrinsic transcripts (CTNNB1, EPCAM, ITGAV), consistent with an immune-excluded or cold tumor phenotype. These changes were compartment-specific and most evident in immune regions rather than tumor areas, underscoring the value of spatial profiling. Wnt signaling via CTNNB1 has been linked to dendritic-cell suppression and immune evasion [35]. Patients with immune-suppressed metastatic nodes may derive limited benefit from ICB monotherapy and could require combinations targeting Wnt, TGF-β, or myeloid programs.

Stratification of metastatic LNs by nodal burden revealed stage-specific immune dysfunction. Although tumor or pseudo-bulk compartments showed no significant differences between N1–2 and N3, immune compartments in N3 exhibited higher ICOSLG, CD40, IFNGR1, and IL6 expression. IL6 and ICOSLG have been implicated in resistance to PD-1 blockade and represent rational targets for combination immunotherapy [36, 37]. Clinically, these markers may identify patients at high risk of recurrence despite nodal clearance and support development of prognostic assays or adjuvant immunotherapy strategies.

Unexpectedly, non-metastatic LNs from N3 patients also exhibited distinct immune alterations. Upregulation of CD74, HLA-DRB, and B2M in the absence of overt metastatic involvement suggests that immune remodeling may occur within tumor-draining LNs before histologically detectable metastasis, potentially reflecting systemic tumor-derived signals. In contrast, non-metastatic LNs from N1–2 patients retained enrichment of MKI67, FOXP3, STAT2, ARG1, and CD27, indicating a distinct immune transcriptional program. Notably, these differences were observed in the subgroup with significantly poorer recurrence-free survival (Fig. 1), supporting the notion that immune alterations extend beyond metastatic deposits and may be associated with advanced nodal disease burden. Together, these findings suggest that profiling histologically uninvolved LNs may provide additional insight into LN immune remodeling and disease progression.

As immunotherapy strategies continue to be explored in gastric cancer, our findings indicate that compartment-specific nodal immune profiling may provide biologic insight for stratifying patients with N1–N3, M0 disease and for rationally designing combination immunotherapeutic approaches.

Bulk and tumor-compartment analyses failed to detect significant differences across metastasis status or N stage, highlighting a key limitation of conventional profiling. Without spatial resolution, immune remodeling is obscured by tumor-dominant signals, arguing for compartment-resolved designs in translational studies.

More broadly, our findings are consistent with accumulating pre-clinical and clinical evidence that demonstrates how tumor-draining LNs undergo substantial immune remodeling during cancer progression [38]. Pre-clinical studies have shown that primary tumors can induce immune suppression, altered antigen presentation, and pre-metastatic niche formation within regional LNs before overt metastatic dissemination [39]. Similarly, clinical profiling studies in breast cancer, melanoma, and colorectal cancer have identified alterations in antigen-presentation pathways, immune checkpoint signaling, inflammatory responses, and immune-cell function within metastatic and non-metastatic LNs [40–42]. Together, these observations support the concept that LNs are active immunological sites rather than passive reservoirs of metastatic cells. Spatial transcriptomic approaches provide a powerful framework for resolving these compartment-specific immune dynamics and linking them to metastatic status and nodal burden [43]. These findings may facilitate the integration of nodal immune profiling into risk stratification and the development of immunotherapy strategies in gastric cancer [44].

This study has limitations. The sample size (*n* = 13) reflects technical demands of spatial transcriptomics and limited availability of well-preserved archival LNs. The focused 84-gene immune panel may not capture broader immune programs. Transcriptomic signals were not validated at the protein level, although key markers such as CD8, PD-1, and ICOSLG warrant confirmation by immunohistochemistry or immunofluorescence. Longer longitudinal follow-up is needed in future studies. In addition, the number of profiled LNs per patient was limited, precluding robust assessment of inter-patient and intra-patient LN heterogeneity. Future studies with larger cohorts and more extensively sampled LNs will be needed to characterize patient-specific spatial immune remodeling. Detailed measurements of LN size and metastatic deposit size were not uniformly available from the pathology records. Although all GeoMx DSP analyses were performed using standardized tissue microarray cores and ROIs, future studies incorporating detailed pathological measurements may provide further insight into the relationship between nodal architecture, metastatic burden, and immune remodeling. Future spatial transcriptomic studies integrating primary tumors, metastatic LNs, and non-metastatic LNs will be valuable for characterizing the spatiotemporal evolution of the metastatic process and the development of pre-metastatic immune remodeling. Although GeoMx DSP enabled compartment-specific spatial profiling, it did not provide single-cell spatial resolution. Future studies using higher-resolution spatial transcriptomic platforms and advanced spatial modeling approaches may further characterize cellular interactions and fine-scale spatial organization within metastatic LNs.

Despite these constraints, we show that immune suppression localizes to immune compartments in metastatic nodes, that higher nodal burden associates with progressive immune dysfunction, and that tumor-free nodes show early remodeling in advanced disease. These insights support exploratory biomarker development, risk stratification, and therapeutic targeting of IL6, ICOSLG, and CD40.

## Conclusions

This study provides a spatial transcriptomic view of metastatic and non-metastatic LNs in gastric adenocarcinoma, revealing compartment-specific immune remodeling associated with metastasis and nodal burden. Non-metastatic nodes retained immune surveillance features, whereas metastatic nodes exhibited immune suppression and tumor-associated transcriptional changes, particularly within immune compartments. Higher nodal burden (N3) was linked to immune exhaustion signatures even in uninvolved nodes. Although immunotherapy is not currently standard for N1–N3, M0 gastric cancer, the LN immune signatures identified here may help guide patient selection and the design of future immunotherapy strategies. Elevated IL6, ICOSLG, and CD40 in high-burden disease represent potential targets for combination or myeloid-directed therapies. Immune alterations in uninvolved nodes support the concept of a pre-metastatic niche and justify their inclusion in biomarker-development efforts. Overall, spatial transcriptomics reveals clinically relevant immune heterogeneity within the lymphatic tumor microenvironment and informs precision immuno-oncology in gastric cancer.

## Supplementary Information

Below is the link to the electronic supplementary material.

Supplementary Material 1

Supplementary Material 2

Supplementary Material 3

Supplementary Material 4

Supplementary Material 5

Supplementary Material 6

## Data Availability

The GeoMx DSP spatial transcriptomic data generated and analyzed in this study are provided in the supplementary file (1) The reporter code count (RCC) files are provided in the supplementary file (2) Additional supporting materials, including Supplementary Tables S1–S3 and Supplementary Figures S1–S3, are available with this article. Additional data are available from the corresponding author upon request.
