# Supplementary material for: Spatial transcriptomics reveals immune remodeling in lymph nodes of gastric adenocarcinoma
Source: Clin Exp Metastasis. 2026 Sep 17;43(5):49. doi: 10.1007/s10585-026-10429-7 (PMC13585797; doi:10.1007/s10585-026-10429-7)
Supplement: Supplementary file 1 [file 10585_2026_10429_MOESM6_ESM.pdf]

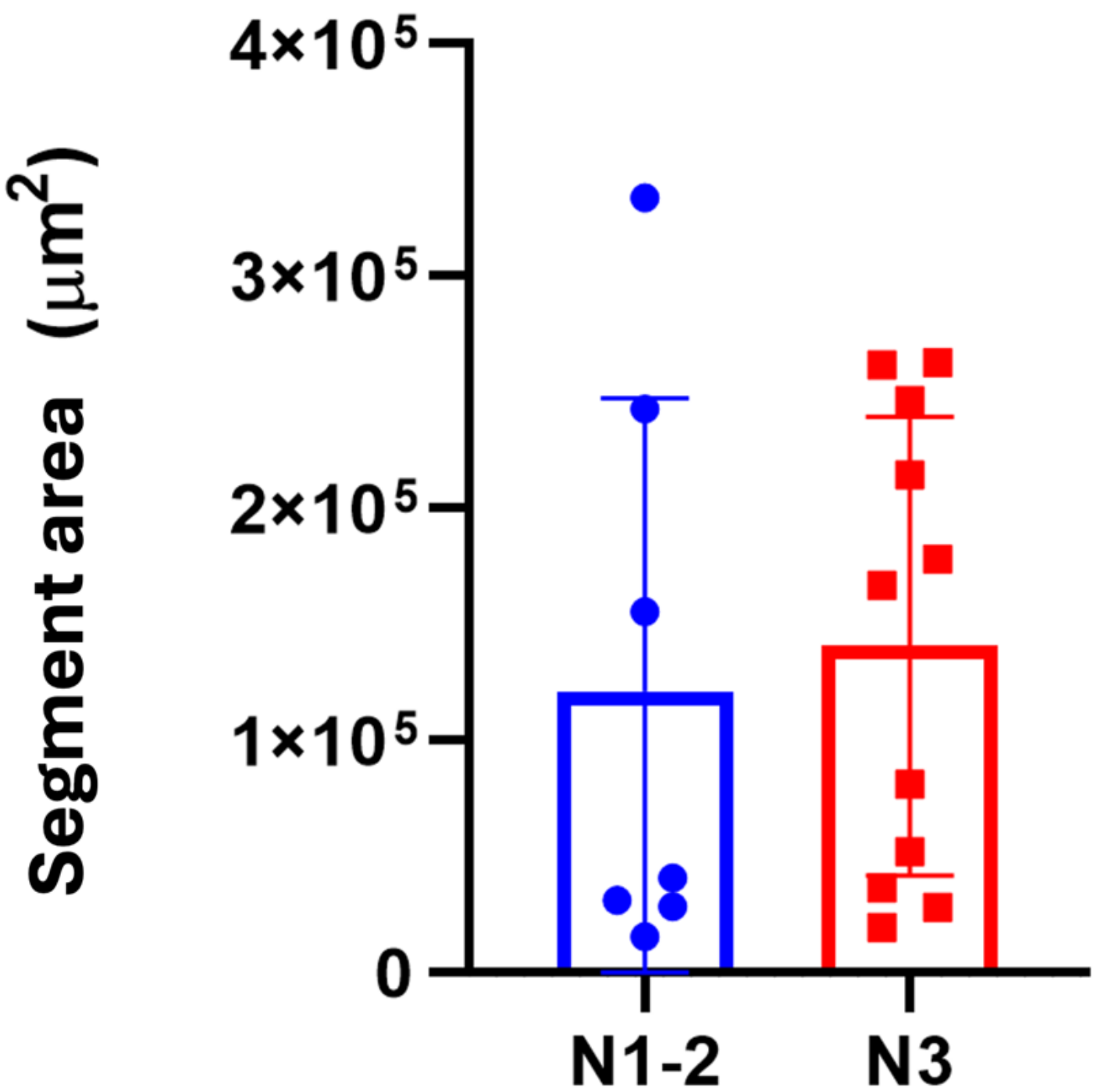

Supplementary Figure S1. Comparison of segmented PanCK-positive area between metastatic lymph nodes from N1–2 and N3 gastric cancer patients. The segmented PanCK-positive area within the profiled regions of interest (ROIs) was quantified to assess potential differences in profiled tumor burden between groups. No significant difference in PanCK-positive area was observed between metastatic lymph nodes from N1–2 and N3 patients (Mann–Whitney U test, *P* = 0.536), suggesting that the transcriptional differences identified in this study were unlikely to be attributable to variation in the size of the profiled tumor compartment.

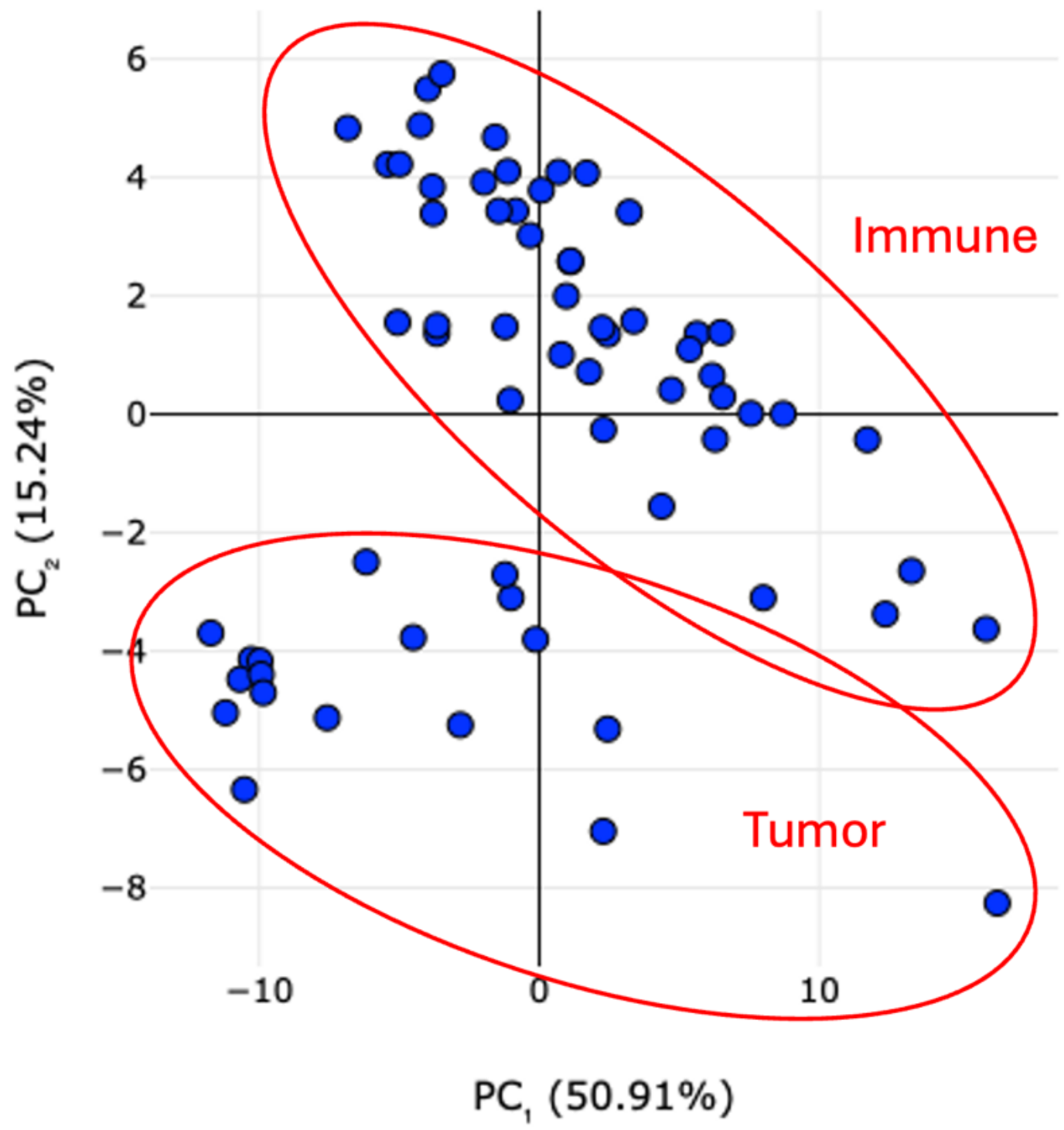

Supplementary Figure S2. Principal component analysis of all profiled GeoMx DSP compartments. Principal component analysis (PCA) was performed on all 65 segmented compartments derived from metastatic and non-metastatic lymph nodes. PC1 and PC2 explained 50.9% and 15.2% of the total variance, respectively. Tumor ($PanCK^{+}$) and immune ($PanCK^{-}$) compartments demonstrated distinct clustering patterns, indicating that the spatial transcriptomic profiles were primarily driven by compartment identity. These findings support the biological separation of tumor and immune microenvironments captured by the GeoMx DSP platform. PCA was performed using normalized GeoMx DSP expression data and visualized using the online PCA tool available at StatsKingdom (https://www.statskingdom.com/pca-calculator.html).

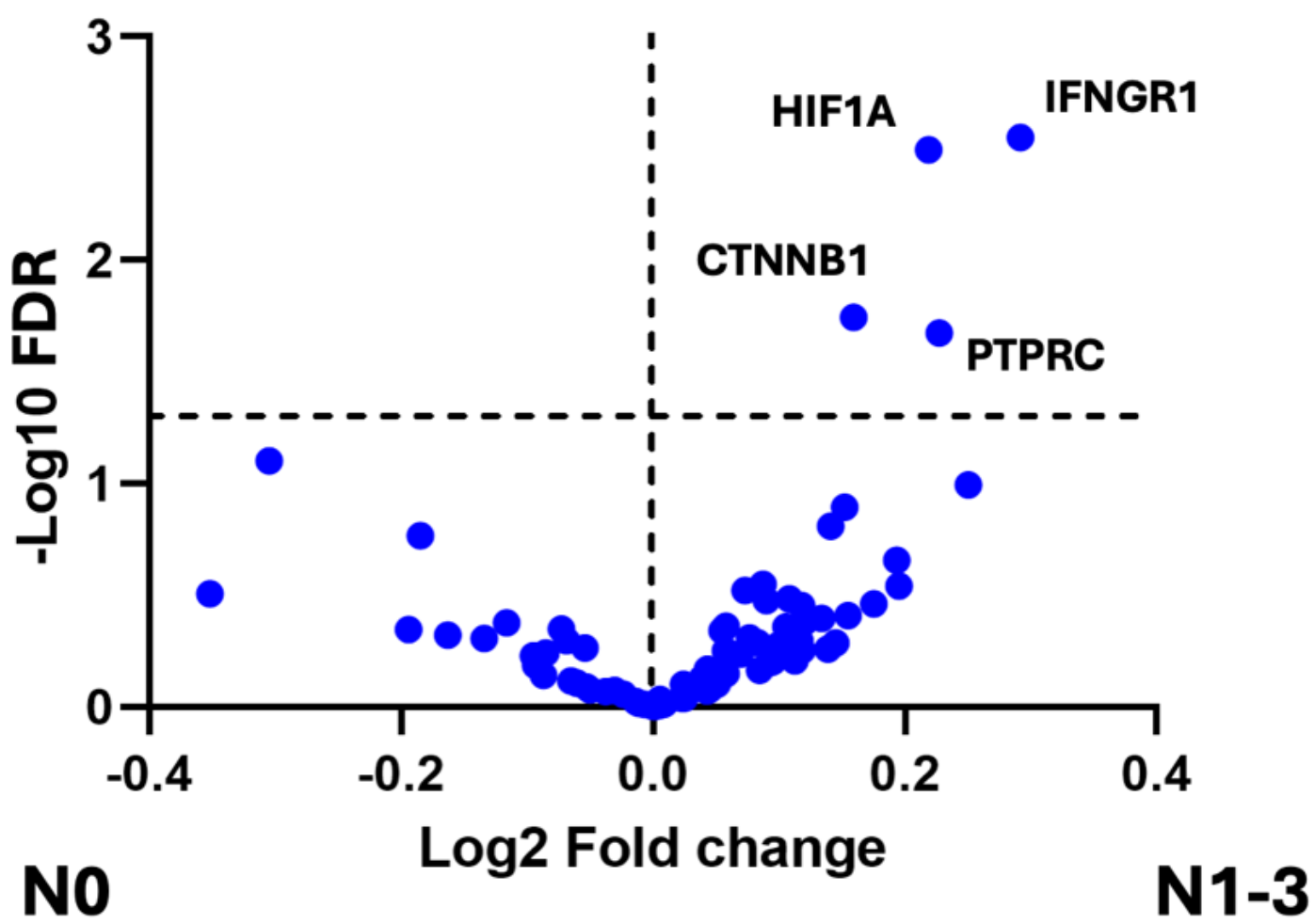

Supplementary Figure S3. Differential gene expression analysis of histologically negative lymph nodes from node-positive (N1–N3, *n* = 13) and node-negative (N0, *n* = 4) gastric cancer patients. Volcano plot showing log2FC (N1–N3/N0) versus −log10(FDR). The horizontal dashed line indicates the significance threshold (FDR = 0.05), and the vertical dashed line indicates no change in expression (log2FC = 0). Four genes, IFNGR1, HIF1A, CTNNB1, and PTPRC, were significantly upregulated in non-metastatic lymph nodes from node-positive patients compared with lymph nodes from node-negative patients. These findings suggest that transcriptional alterations may already be present in tumor-draining lymph nodes prior to histologically detectable metastatic involvement, supporting the concept of early nodal immune remodeling. Abbreviations: FDR, false discovery rate; FC, fold change; LN, lymph node.
